# Dynamic Chronological Changes in Serum Triglycerides Are Associated With the Time Point for Non-alcoholic Fatty Liver Disease Development in the Nationwide Korean Population Cohort

**DOI:** 10.3389/fmed.2021.637241

**Published:** 2021-03-10

**Authors:** John Hoon Rim, Taemi Youk, Heon Yung Gee, Jooyoung Cho, Jongha Yoo

**Affiliations:** ^1^Department of Laboratory Medicine, Yonsei University College of Medicine, Severance Hospital, Seoul, South Korea; ^2^Department of Medicine, Physician-Scientist Program, Yonsei University Graduate School of Medicine, Seoul, South Korea; ^3^Department of Pharmacology, Yonsei University College of Medicine, Seoul, South Korea; ^4^Research Institute, National Health Insurance Service Ilsan Hospital, Goyang, South Korea; ^5^Department of Statistics, Korea University, Seoul, South Korea; ^6^Department of Laboratory Medicine, Wonju Severance Christian Hospital, Yonsei University Wonju College of Medicine, Wonju, South Korea; ^7^Department of Laboratory Medicine, National Health Insurance Service Ilsan Hospital, Goyang, South Korea

**Keywords:** non-alcoholic fatty liver disease, laboratory results, lifestyle factors, serum triglyceride, nationwide study

## Abstract

**Background:** We investigated the effects of anthropometric, laboratory, and lifestyle factors on the development of non-alcoholic fatty liver disease (NAFLD) in a nationwide, population-based, 4-year retrospective cohort.

**Methods:** The propensity score-matched study and control groups contained 1,474 subjects who had data in the Korean National Health Insurance Service-National Sample Cohort in 2009, 2011, and 2013. NAFLD was defined using medical records of a diagnosis confirmed by primary clinicians and meeting two previously validated fatty liver prediction models. Chronological changes in anthropometric variables, laboratory results, and lifestyle factors during two periods were compared between patient and control groups in order to find out parameters with consistent dynamics in pre-NAFLD stage which was defined as period just before the NAFLD development.

**Results:** Among the 5 anthropometric, 10 laboratory, and 3 lifestyle factors, prominent chronological decremental changes in serum triglycerides were consistently observed during the pre-NAFLD stage, although the degrees of changes were more predominant in men (−9.46 mg/dL) than women (−5.98 mg/dL). Furthermore, weight and waist circumference changes during the pre-NAFLD stage were noticeable only in women (+0.36 kg and +0.9 cm for weight and waist circumference, respectively), which suggest gender difference in NAFLD.

**Conclusion:** Early screening strategies for people with abrupt chronological changes in serum triglycerides to predict NAFLD development before the progression is recommended.

## Introduction

Non-alcoholic fatty liver disease (NAFLD), a disease complex spectrum ranging from benign hepatic steatosis to hepatic inflammation, fibrosis, and cirrhosis, is now the leading cause of chronic liver disease in developed countries (1). In Korea, the prevalence of fatty liver is estimated to be as high as 25–30% (2), and 10–15% of those affected could have steatohepatitis with inflammation. Because metabolic syndrome components such as obesity and diabetes are independent predictors of NAFLD (3), many reports emphasize comprehensive lifestyle modifications based on reduced energy intake and increased physical activity as a primary therapeutic intervention for NAFLD (4–7). Although systematic guidelines for NAFLD assessment and management proposed by the National Institute for Health and Care Excellence (NICE) recommend ultrasonography as the first-line diagnostic step and lifestyle intervention for management of NAFLD (8), few publication has previously reported data on changes in NAFLD-associated parameters from a chronological viewpoint (9), and none using real-world human clinical data.

As the early detection of NAFLD becomes more important, identification of early changes that predict future NAFLD development and chronological changes in parameters evaluated in the national health screening examination is essential to both the clinical field and national policy. Korea has systematic and comprehensive national registry data collected from the Korean National Health Insurance Service-National Sample Cohort (KNHIS-NSC) (10). The KNHIS-NSC database is a population-based sample cohort. Its purpose is to provide representative, useful health insurance and health examination data to public health researchers and policymakers. A total of 1,025,340 participants (513,258 men and 512,082 women), 2.2% of the total eligible population in the 2002 Korean nationwide health insurance database, were selected using proportionally allocated, stratified, systematic random sampling with a total medical expenses distribution within strata reflecting gender, age group, qualifications, and income quintile. Importantly, the data include laboratory results from general health examinations of cohort participants that were followed for 11-years, until 2013.

The purpose of this study was to investigate the effects of anthropometric, laboratory, and lifestyle factors recorded in the KNHIS-NSC database on the development of NAFLD in a nationwide, population-based, 4-year retrospective cohort.

## Materials and Methods

### Database

In Korea, all citizens are obligated to enroll in the KNHIS. A total of 97% of the Korean population is covered by the Medical Assistance Program, and 3% of the Korean population is covered by the Medical Care for Patriots and Veterans Affairs Scheme, respectively. Thus, nearly all of the data in the health system are centralized in large databases. In Korea, patients with KNHIS pay ~30% of their total medical expenses, and medical providers are required to submit claims for the remaining 70%. Claims are accompanied by data regarding diagnostic codes, procedures, prescription drugs, personal information about the patient, information about the hospital, the direct medical costs of both inpatient and outpatient care, and dental services. No health care records are duplicated or omitted because all Korean residents receive a unique identification number at birth for use by the Korean government for purposes related to the health care system. For diagnostic codes, the KNHIS uses the Korean Classification of Diseases (KCD), which is similar to the International Classification of Diseases (ICD).

This study adhered to the tenets of the Declaration of Helsinki, and the KNHIS-NSC 2002–2013 project was approved by the Institutional Review Board of the KNHIS. This study design was reviewed and approved by the Institutional Review Board of the National Health Insurance Service, Ilsan Hospital (IRB No. 2015-11-010). Written informed consent was waived.

### Study Population

Data for this study were derived from the KNHIS-NSC 2009–2013. This database includes all medical claims and general health examination results filed from January 2009 to December 2013. The study population for this study was nationally representative subjects who have undertaken annual medical check-ups in all three of the following years: 2009, 2011, and 2013.

**Figure 1:**
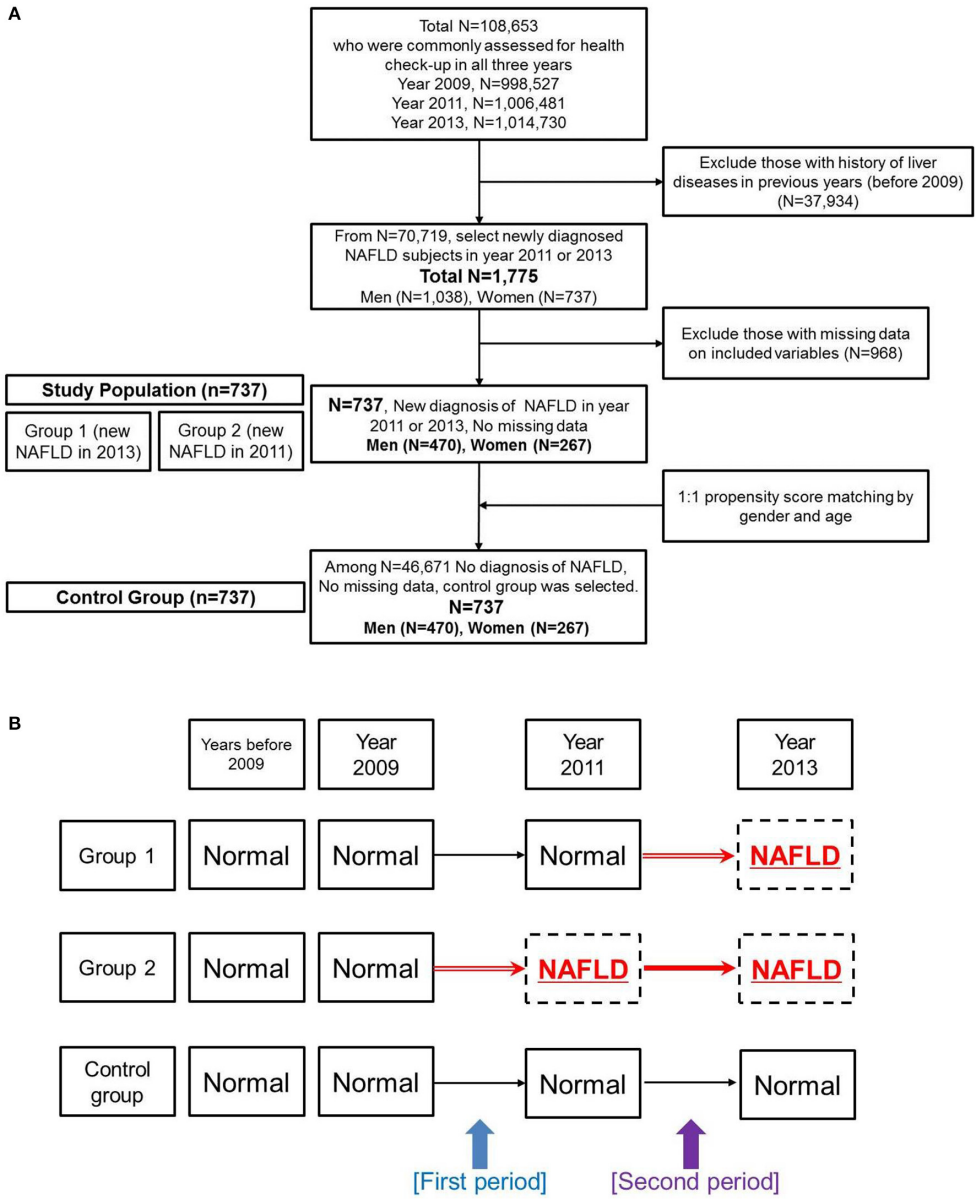
Overview of the study design. **(A)** Flowchart of study population selection. **(B)** Designations for subgroups of the non-alcoholic fatty liver disease group and for the control group. Changes were evaluated during two periods.

A flowchart for the study population is in Figure 1A. From a total of 108,653 Koreans who participated in medical check-ups in all 3-years (2009, 2011, and 2013), we excluded subjects with a history of liver disease before 2009 (*n* = 37,934) using the KNHIS-NSC 2002–2008 database. Then, we selected individuals who were newly diagnosed with NAFLD in 2011 or 2013 (The criteria for NAFLD diagnosis is explained in section Definition of NAFLD). Among the 1,775 subjects newly diagnosed with NAFLD in 2011 or 2013, we excluded those missing anthropometric, biochemical, medical history, or lifestyle variables that we examined in this study (*n* = 968). Thus, we analyzed a total of 737 NAFLD patients (470 men and 267 women) for this study. We derived a control group (470 men and 267 women) from the whole population without an NAFLD diagnosis (*n* = 46,671) using a propensity score matching algorithm for age and gender. All subjects in the control group received negative results when evaluated by the hepatic steatosis index and fatty liver index using laboratory results from 2009, 2011, and 2013.

We further divided the NAFLD group into subgroups (Figure 1B). Group 1 was defined as subjects who did not have a diagnosis of NAFLD in 2009 or 2011 but who had newly developed NAFLD in 2013. Group 2 was defined as subjects who did not have a diagnosis of NAFLD in 2009 but showed newly developed NAFLD in 2011 and sustained the diagnosis of NAFLD in 2013.

### Study Period

Because time-dependent changes in various factors were the major end-point result, we assigned chronological differences from 2009 to 2011 as the first period and chronological differences from 2011 to 2013 as the second period (Figure 1B). “Pre-NAFLD stage” was defined as the period just before the NAFLD development for patient groups. Therefore, the first period was the pre-NAFLD stage for group 2, and the second period was the pre-NAFLD stage for group 1. Although the data did not contain the exact time when NAFLD developed in each patient, we assumed a 2-year difference for both the first and the second periods as an approximate value.

### Definition of NAFLD

Each patient was tracked on the basis of his or her index dates for ambulatory and inpatient care visits over the 11-years from 2003 to 2013, to detect those patients who developed NAFLD (KCD code K76.0, corresponding to ICD-10-CM code K76.0). To rule out the effects of liver disease with other etiologies, we excluded subjects who also had a diagnosis of alcoholic fatty liver or toxic liver (KCD codes K70–75).

To confirm the medical diagnosis of NAFLD as assessed by patients' primary physicians, we calculated two previously validated fatty liver prediction models: (1) hepatic steatosis inde × (HSI) = 8 × [alanine transferase (ALT)/aspartate transferase (AST) ratio] + body mass index (BMI) (+2, if female; +2, if diabetes mellitus) (11) and (2) fatty liver index (FLI) = [[e∧ [0.953 × loge [triglycerides (TG)] + 0.139 × BMI + 0.718 × loge [gamma-glutamyl transferase (GGT)] + 0.053 × waist circumference −15.745]]]/[[1+e∧ [0.953 × loge (TG) + 0.139 × BMI + 0.718 × loge (GGT) + 0.053 × waist circumference −15.745]]] × 100, with TG measured in mg/dL, GGT in U/L, and waist circumference in cm (12). All parameters for the equations were available in the database. We defined NAFLD as HSI of 35 or higher or FLI of 60 or higher, following a previous study (13).

### Anthropometric, Laboratory, and Lifestyle Variables

We chronologically compared 18 variables in all subjects in the 3 groups, calculating absolute differences of mean values for numerical parameters and percentage differences for categorical parameters from 2009 to 2011 and from 2011 to 2013. As the time intervals were both ~2-years, we could also compare changes between 2 time periods within each group.

For anthropometric factors, we evaluated body weight, BMI, systolic blood pressure (SBP), diastolic blood pressure (DBP), and waist circumference. Body weight was measured to the nearest 0.1 kg. BMI was calculated as weight/height^2^ (kg/m^2^). Waist circumference was measured at the narrowest point between the lower border of the rib cage and the iliac crest.

For laboratory values, we measured a total of 10 parameters in blood samples: liver enzymes (AST, ALT, and GGT), fasting glucose, total cholesterol, TG, high-density lipoprotein (HDL) cholesterol, low-density lipoprotein (LDL) cholesterol, creatinine in serum samples, and hemoglobin level in whole blood sample.

For the lifestyle evaluation, we reviewed questionnaires related to smoking, alcohol drinking, and exercise status. Because all questions were answered based on each individual's experience, we objectively categorized answers into binary outcomes for smoking (current smoking or not) and exercise (no exercise at all or at least minimal physical activity). For alcohol consumption, we scored an alcohol index according to the following criteria: 0 = no consumption, 1 = alcohol fewer than or equal to 2 days per week, 2 = alcohol more than 3 days and fewer than or equal to 4 days per week, 3 = alcohol more than 5 days per week.

For the family history investigation, baseline profiling on hypertension, stroke, cardiovascular disease, and diabetes mellitus were incorporated for only available participants based on self-check questionnaires.

### Statistical Methods

Participants' characteristics were compared using independent-sample Student's *t*-tests for continuous variables and Chi-square tests for categorical variables. The differences between the 2 NAFLD subgroups and control group were verified by ANOVA test. All data are presented as mean ± standard deviation for continuous variables and frequency percentages for categorical variables. *P* < 0.05 were considered statistically significant. All analyses were conducted using SAS v9.4 (SAS Institute Inc., USA).

## Results

### Characteristics of the Study Population

The clinical features of the study population at baseline (2009) are shown in Table 1. Because propensity score matching was based on age group and gender, the NAFLD group and control group were the same in terms of age group distribution and gender percentages. All tested anthropometric factors were statistically significantly higher in the NAFLD group than in the control group, with the exception of height. Considering the laboratory variable measurements, all three liver enzymes showed statistically significant differences between the NAFLD group and the control group in both genders, as expected. Interestingly, serum HDL cholesterol level was lower in the NAFLD group than in the control group with statistical significance in men but without statistical significance in women. Additionally, the NAFLD group showed higher LDL cholesterol than the control group with statistical significance in women but without statistical significance in men. For the lifestyle parameters, we found no statistically significant differences in either gender for any variable (smoking, alcohol consumption, or exercise status). For the family history variables, men in the NAFLD group appeared to report more family history of hypertension and diabetes mellitus compared to the control group, which was not observed in women.

**Table 1:** Clinical characteristics of study population measured in year 2009 as baseline characteristics.

**Gender**	**Male**			**Female**		
	**NAFLD**	**Normal**	***p*-value**	**NAFLD**	**Normal**	***p*-value**
	**(*N* = 470)**	**(*N* = 470)**		**(*N* = 267)**	**(*N* = 267)**	
**DEMOGRAPHIC FACTORS [NUMBER, (%)]**						
**Age group (years)**						
20–29	35 (7.4)	35 (7.4)	1.00	1 (0.4)	1 (0.4)	1.00
30–39	128 (27.2)	128 (27.2)		10 (3.7)	10 (3.7)	
40–49	152 (32.3)	152 (32.3)		56 (21.0)	56 (21.0)	
50–59	91 (19.4)	91 (19.4)		115 (43.1)	115 (43.1)	
60–69	51 (10.9)	51 (10.9)		64 (24.0)	64 (24.0)	
>70	13 (2.8)	13 (2.8)		21 (7.9)	21 (7.9)	
**ANTHROPOMETRIC FACTORS [MEAN, (SD)]**						
Height (cm)	170.5 (6.4)	170.0 (5.8)	0.2408	155.0 (5.6)	155.3 (5.9)	0.5550
Weight (kg)	78.6 (9.7)	66.2 (7.7)	< .0001	64.5 (7.7)	54.1 (5.4)	< .0001
Waist circumference (cm)	90.1 (7.2)	80.5 (6.1)	< .0001	84.9 (7.9)	75.0 (6.4)	< .0001
Body mass index (kg/m^2^)	27.0 (2.8)	22.9 (2.1)	< .0001	26.8 (2.7)	22.5 (2.0)	< .0001
Systolic blood pressure (mmHg)	127.1 (13.3)	123.6 (13.0)	< .0001	126.8 (14.5)	122.5 (16.0)	0.0012
Diastolic blood pressure (mmHg)	80.2 (9.3)	77.2 (9.0)	< .0001	77.9 (9.2)	75.2 (9.5)	0.0008
**LABORATORY RESULTS [MEAN, (SD)]**						
Hemoglobin (g/dl)	15.3 (1.2)	14.9 (1.1)	< .0001	13.0 (1.3)	12.8 (1.0)	0.0302
Fasting glucose (mg/dL)	103.7 (26.7)	95.7 (20.2)	< .0001	101.1 (25.2)	93.6 (15.9)	< .0001
Total cholesterol (mg/dL)	202.3 (36.8)	192.1 (34.0)	< .0001	208.0 (41.9)	199.4 (36.9)	0.0127
Triglycerides (mg/dL)	196.4 (125.6)	134.7 (101.9)	< .0001	143.7 (81.2)	108.4 (67.8)	< .0001
HDL cholesterol (mg/dL)	49.0 (11.1)	55.1 (22.5)	< .0001	58.5 (61.9)	60.6 (43.7)	0.6575
LDL cholesterol (mg/dL)	115.1 (46.6)	111.1 (31.4)	0.1302	125.5 (37.2)	119.3 (33.9)	0.0456
Serum creatinine (mg/dL)	1.4 (2.0)	1.4 (2.0)	0.8944	0.9 (0.8)	0.9 (0.7)	0.8474
AST (U/L)	33.1 (16.3)	24.8 (19.6)	< .0001	30.2 (30.6)	23.4 (13.1)	0.001
ALT (U/L)	50.8 (32.7)	21.8 (9.7)	< .0001	37.3 (44.7)	17.4 (8.7)	< .0001
GGT (U/L)	71.0 (67.4)	36.0 (29.2)	< .0001	36.2 (53.8)	18.3 (10.2)	< .0001
FAMILY HISTORY^*^ **[NUMBER, (%)]**						
Hypertension	74 (22.6)	27 (8.3)	< .0001	36 (21.6)	34 (20.7)	0.8542
Stroke	44 (13.5)	30 (9.3)	0.0917	24 (14.4)	24 (14.8)	0.9093
Cardiovascular disease	16 (4.9)	7 (2.2)	0.059	14 (8.4)	7 (4.3)	0.1318
Diabetes mellitus	59 (18.0)	31 (9.6)	0.0018	30 (18.0)	22 (13.5)	0.2655
**LIFE STYLE FACTORS [NUMBER, (%)]**						
**Smoke**			0.5538			0.0798
No	261 (55.5)	270 (57.4)		264 (98.9)	258 (96.6)	
Current smoker	209 (44.5)	200 (42.6)		3 (1.1)	9 (3.4)	
**Alcohol consumption (days/a week)**			0.1812			0.4846
0: 0	152 (32.3)	137 (29.1)		218 (81.6)	222 (83.1)	
1–2: 1	220 (46.8)	244 (51.9)		44 (16.5)	39 (14.6)	
3–4: 2	77 (16.4)	61 (13.0)		5 (1.9)	4 (1.5)	
>5: 3	21 (4.5)	28 (6.0)		0 (0.0)	2 (0.7)	
**Exercise**			0.1691			0.2552
No	90 (19.1)	74 (15.7)		73 (27.3)	85 (31.8)	
Yes (at least little physical activity)	380 (80.9)	396 (84.3)		194 (72.7)	182 (68.2)	

* *Participants with family history information available were only included in the analysis*.

*NAFLD, Non-alcoholic fatty liver disease; SD, standard deviation; HDL, high-density lipoprotein; LDL, low-density lipoprotein; AST, aspartate aminotransferase; ALT, alanine aminotransferase; GGT, gamma-glutamyl transferase*.

### Chronological Changes in Anthropometric and Laboratory Parameters in the NAFLD Subgroups

**Figure 2:**
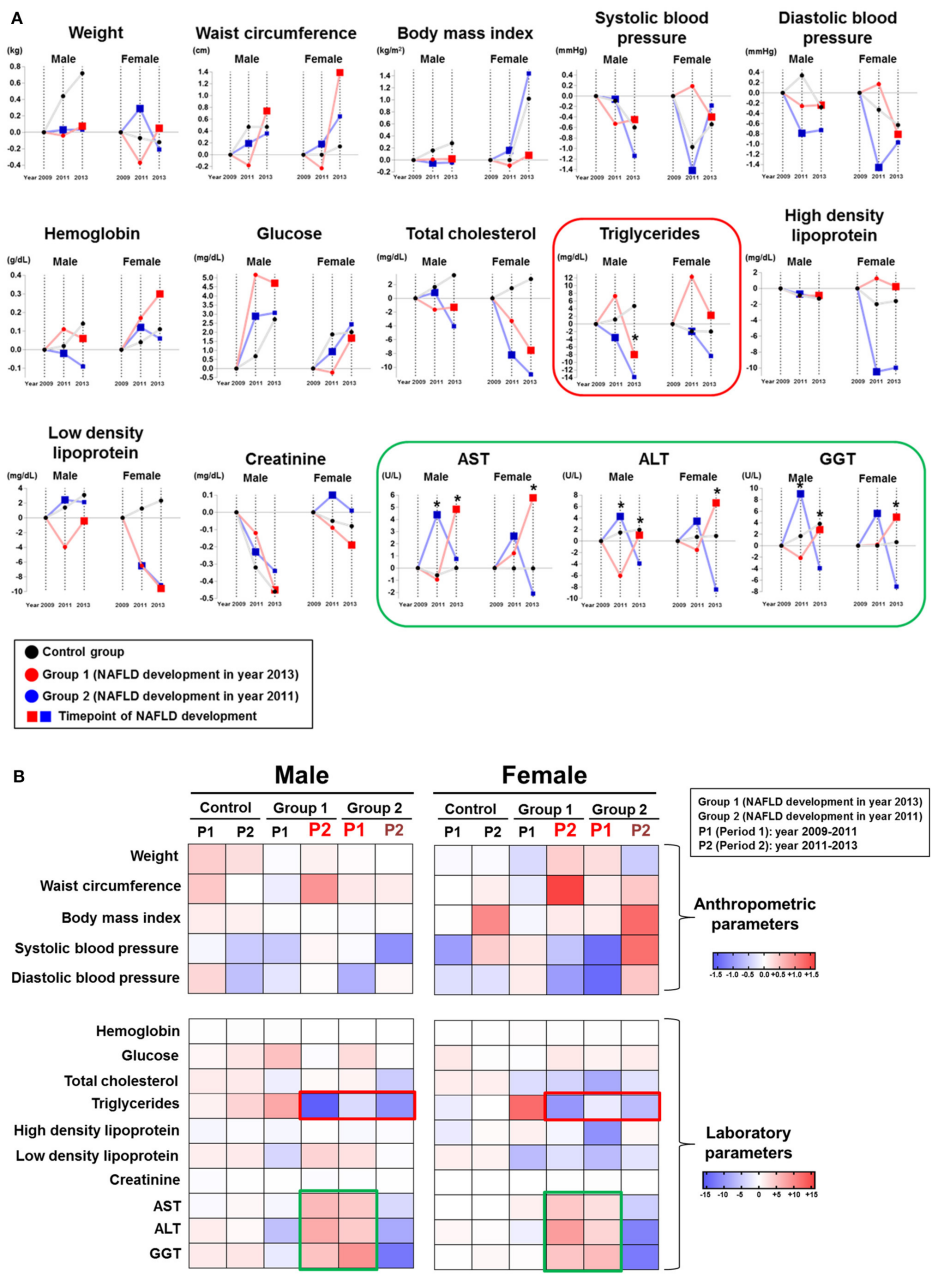
Chronological changes of anthropometric and laboratory parameters according to NAFLD development group and gender. **(A)** Chronological changes of incremental and decremental changes are presented. **(B)** Delta difference values of each parameter during two periods are presented (Group 1: patients who newly developed NAFLD in year 2013; Group 2: patients who newly developed NAFLD in year 2011).

We compared the time-dependent changes in anthropometric and laboratory parameters by absolute mean value differences among the 2 NAFLD subgroups and the control group by gender (Figure 2). When liver enzyme dynamics during pre-NAFLD stage were evaluated, AST, ALT, and GGT all increased in group 2 during the first period (2009–2011) with statistical significance in men, but not in women. Furthermore, during the second period (2011–2013), AST, ALT, and GGT all increased in group 1 and decreased in group 2 with statistical significance in both genders, as expected (Figure 2, green box).

Importantly, serum TG in both group 1 and group 2 showed the most prominent decrease during pre-NAFLD stages in both genders (Figure 2, red box). Interestingly, the decremental change of serum TG in men for group 2 during pre-NAFLD stage was most prominent with statistical significance (*p* < 0.05). The average degree of decrease in serum TG level among NAFLD patient groups compared to control group in pre-NAFLD stages was 8.38 mg/dL. Additionally, total cholesterol in females showed the most significant decrease in group 2 during the first period and in group 1 during the second period, although statistical significance was observed only in the first period.

Among the anthropometric factors, weight and waist circumference showed incremental change during pre-NAFLD stage before NAFLD was newly diagnosed (first period for group 2 and second period for group 1) but only in women. SBP and DBP decreased more in group 1 during the second period and in group 2 during the first period in women, although without statistical significance. In men, parameters showed variable changes in the pre-NAFLD stage periods for the NAFLD group compared to the control group.

### Chronological Changes in Lifestyle Factors in the NAFLD Subgroups

Among the three lifestyle factors considered in this study, we observed different patterns by gender (Table 2). In men, we found no consistent chronological change in pre-NAFLD stage periods for both groups. In contrast, the proportion of females with no exercise increased in group 2 during the first period (+1.46 %) and in group 1 during the second period (+0.77%). In other words, more NAFLD was diagnosed in female subjects who did not perform any exercise. Furthermore, the alcohol consumption scores in females decreased in greater degree in group 1 during the second period (−0.11) compared to other groups in the corresponding periods, while men did not show similar consistent trends.

**Table 2:** Chronological changes of lifestyle factors in study subgroups.

**Gender**	**Male**						**Female**					
**Period**	**From 2009 to 2011 (first period)**			**From 2011 to 2013 (second period)**			**From 2009 to 2011 (first period)**			**From 2011 to 2013 (second period)**		
**Group**	**NAFLD**		**Normal**	**NAFLD**		**Normal**	**NAFLD**		**Normal**	**NAFLD**		**Normal**
	**Group 1**	**Group 2**	**(*N* = 470)**	**Group 1**	**Group 2**	**(*N* = 470)**	**Group 1**	**Group 2**	**(*N* = 267)**	**Group 1**	**Group 2**	**(*N* = 267)**
	**(*N* = 228)**	**(*N* = 242)**		**(*N* = 228)**	**(*N* = 242)**		**(*N* = 130)**	**(*N* = 137)**		**(*N* = 130)**	**(*N* = 137)**	
**LIFE STYLE FACTORS**												
Current smoker, %p	−1.32	−4.58	−3.40	−8.89	−2.92	−1.92	0.00	0.00	0.00	0.77	0.00	0.00
Alcohol consumption score	0.11	0.04	−0.02	−0.10	−0.02	0.01	0.09	−0.01	0.02	−0.11	−0.01	−0.02
No exercise, %p	−8.33	1.24	4.04	0.88	1.24	−2.35	0.77	1.46	−7.52	0.77	−1.46	0.38

*NAFLD, Non-alcoholic fatty liver disease*.

## Discussion

In this study, we evaluated the effects of 18 anthropometric, laboratory, and lifestyle factors on the development of NAFLD by comparing chronological changes in parameters in association with new NAFLD diagnoses. Given a retrospective cohort study population, we could derive chronological changes for each subject across a 4-year period using the consistent approach of national health check-ups. While previous studies in various populations focused on finding risk factors associated with NAFLD in a cross-sectional study setting (14), we could reveal several interesting findings for associations between NAFLD and changes in measured factors over time, focusing on dynamics in pre-NAFLD stages according to gender.

Firstly, it is interesting that the serum TG level, which is normally highlighted for its important role in hyperlipidemia and metabolic syndrome when increased, is inversely associated with NAFLD, especially in men. Importantly, the decremental changes of serum TG level in pre-NAFLD stage were previously suggested as a potential biomarker to identify critical transition state for NAFLD in animal model (9). In human studies, a decrease in serum TG level is generally reported to be associated with liver cirrhosis (15). It is plausible that the dynamics of lipid species in serum and liver might be different according to disease progression, especially in pre-NAFLD stage. Although serum lipid profiles were previously studied for their associations with NAFLD (16, 17), chronological changes in liver and serum according to different NAFLD stages should be investigated using traditional parameters as well as up-to-date metabolomics approach (18).

Secondly, the relationship of weight and waist circumference with development of NAFLD appeared to be stronger in women than men. Interestingly, one Japanese population study also showed the similar results that the same degree of weight gain increased the relative risk for NAFLD more prominently in women than in men (19). Several researchers have also reported that the association between obesity and inflammatory markers is considerably stronger in women than men (20, 21), which eventually provoke increased inflammation of hepatocytes leading to NAFLD. Based on previous studies highlighting gender differences of NAFLD (22), further studies with inflammatory marker measurements would prove the hypothesis that women might be more susceptible than men to the inflammatory effects of central obesity and even development of NAFLD.

Lastly, the effects of exercise on the development of NAFLD revealed to be stronger in women than men, which is in line with the previous finding. Many researchers and the NICE guideline emphasize the importance of exercise for NAFLD management and control (8, 23). Our results revealed the greatest increases in sedentary lifestyle among females in the NAFLD patients during the time period of a new diagnosis of NAFLD, the pre-NAFLD stage. Although central obesity could be a main reason for NAFLD development, the degree of physical activity as a lifestyle risk factor for the development of NAFLD should also be emphasized (5, 6).

The fundamental limitation of our study is selection bias associated with any population-based retrospective cohort. Therefore, we were unable to discern direct causal relationships between the various factors and NAFLD. However, this is a large propensity score-matched population study, which promotes the statistical reliability of the results. Additionally, correlation analyses and comparative association quantification among parameters would validate the association of dynamic changes with NAFLD development, which was not performed in this study. Another limitation is associated with diagnosis, because we did not include the current standard diagnostic procedure of a liver biopsy which might provide NAFLD severity level for fibrosis. Even though we used several types of diagnostic parameters, such as HIS and FLI, to increase the confirmatory degree of NAFLD diagnosis, subjective diagnoses dependent on different physicians' medical decisions could still have affected the analysis, as could code input errors in the database.

In conclusion, we investigated chronological changes in anthropometric factors, laboratory results, and lifestyle factors to predict the development of NAFLD during pre-NAFLD stage. Based on our finding of inversely decreasing pattern in serum triglycerides during the pre-NAFLD stage, we suggest early screening strategies for people with abrupt chronological changes in serum triglycerides to predict NAFLD development before the progression. Furthermore, people with abrupt and prominent chronological changes in specific parameters, especially waist circumference and exercise degree for women, might require more detailed follow-up evaluations. A better understanding of the various factors related to NAFLD development will provide insight into preventive interventions to improve health and reduce the incidence of NAFLD-associated disorders.

## Data Availability Statement

As the original contributions presented in the study are included in the article, further inquiries can be directed to the corresponding author/s.

## Ethics Statement

The studies involving human participants were reviewed and approved by The Institutional Review Board of the National Health Insurance Service, Ilsan Hospital (IRB No. 2015-11-010). The patients/participants provided their written informed consent to participate in this study.

## Author Contributions

JHR conducted the research, performed the statistical analysis, and wrote the draft. TY performed the statistical analysis and collected the data. HYG and JC contributed to the literature search and proofreading of the manuscript. JY designed the study and revised the manuscript. All authors read and approved the submitted version.

## Conflict of Interest

The authors declare that the research was conducted in the absence of any commercial or financial relationships that could be construed as a potential conflict of interest.
